# Where is “policy” in dissemination and implementation science? Recommendations to advance theories, models, and frameworks: EPIS as a case example

**DOI:** 10.1186/s13012-022-01256-x

**Published:** 2022-12-12

**Authors:** Erika L. Crable, Rebecca Lengnick-Hall, Nicole A. Stadnick, Joanna C. Moullin, Gregory A. Aarons

**Affiliations:** 1 Department of Psychiatry, University of California, La Jolla, San Diego, CA 92093 USA; 2 Child and Adolescent Services Research Center, San Diego, CA USA; 3 UC San Diego Altman Clinical and Translational Research Institute Dissemination and Implementation Science Center, La Jolla, San Diego, CA USA; 4 The Brown School, Washington University in St. Louis, St. Louis, MO USA; 5 Faculty of Health Sciences, enAble Institute, Curtin University, Perth, WA Australia

**Keywords:** Theory, Model, Framework, Policy, Politics, Context

## Abstract

**Background:**

Implementation science aims to accelerate the public health impact of evidence-based interventions. However, implementation science has had too little focus on the role of health policy — and its inseparable politics, polity structures, and policymakers — in the implementation and sustainment of evidence-based healthcare. Policies can serve as determinants, implementation strategies, the evidence-based “thing” to be implemented, or another variable in the causal pathway to healthcare access, quality, and patient outcomes. Research describing the roles of policy in dissemination and implementation (D&I) efforts is needed to resolve persistent knowledge gaps about policymakers’ evidence use, how evidence-based policies are implemented and sustained, and methods to de-implement policies that are ineffective or cause harm. Few D&I theories, models, or frameworks (TMF) explicitly guide researchers in conceptualizing where, how, and when policy should be empirically investigated. We conducted and reflected on the results of a scoping review to identify gaps of existing Exploration, Preparation, Implementation, and Sustainment (EPIS) framework-guided policy D&I studies. We argue that rather than creating new TMF, researchers should optimize existing TMF to examine policy’s role in D&I. We describe six recommendations to help researchers optimize existing D&I TMF. Recommendations are applied to EPIS, as one example for advancing TMF for policy D&I.

**Recommendations:**

(1) Specify dimensions of a policy’s function (policy goals, type, contexts, capital exchanged).

(2) Specify dimensions of a policy’s form (origin, structure, dynamism, outcomes).

(3) Identify and define the nonlinear phases of policy D&I across outer and inner contexts.

(4) Describe the temporal roles that stakeholders play in policy D&I over time.

(5) Consider policy-relevant outer and inner context adaptations.

(6) Identify and describe bridging factors necessary for policy D&I success.

**Conclusion:**

Researchers should use TMF to meaningfully conceptualize policy’s role in D&I efforts to accelerate the public health impact of evidence-based policies or practices and de-implement ineffective and harmful policies. Applying these six recommendations to existing D&I TMF advances existing theoretical knowledge, especially EPIS application, rather than introducing new models. Using these recommendations will sensitize researchers to help them investigate the multifaceted roles policy can play within a causal pathway leading to D&I success.

**Supplementary Information:**

The online version contains supplementary material available at 10.1186/s13012-022-01256-x.

Contributions to the literature

- We argue that the important role of health policy in dissemination and implementation efforts is understudied, and that more research in this area is needed to accelerate the public health impact of dissemination and implementation efforts.
- We provide six recommendations, exemplified in diverse scenarios, to help researchers conceptualize health policy’s role(s) in dissemination and implementation research.
- We identify potential construct adaptations that will augment theories, models, and frameworks for specifying the role of policy, politics, and polity in implementation efforts.
- Applied recommendations demonstrate how the EPIS framework can be advanced to guide meaningful health policy D&I research.

## Background

Health policies, including the laws, regulations, and administration actions of governmental, public, and private organizations, play a critical role in influencing healthcare access [1], quality [2], and patient and public health outcomes [3], yet the role of policy in dissemination and implementation science (D&I) efforts is often understated or ignored [4, 5]. D&I research frequently conceptualizes health policy as a distal environmental factor within a broadly defined outer context rather than as central to the research and as a target of D&I strategies [6]. Health policies can serve as implementation strategies that facilitate or mandate access to evidence-based health services [7–9]. Health policies can also represent the evidence-based law, rule, or “thing” [10] at the center of implementation efforts [5, 11–14], act as determinants that enable or constrain D&I strategies from achieving desired outcomes [5, 15–17], and potentially serve in other causal pathway roles (e.g., mechanisms).

Meaningful conceptualization of the multifaceted roles that policy plays in D&I research is critical to resolving persistent knowledge gaps about when, how, and why policymakers use evidence to inform policy, how evidence-based policies are implemented and sustained, and methods to de-implement policies that can harm individuals or society. Policy D&I can identify bidirectional roles of actors in the outer (e.g., policymakers) and inner context (e.g., healthcare delivery organizations) that can impact policy discourse and decision-making. Specifying health policy’s role in D&I research will improve empirical measurement of policy-related variables, can accelerate the impact of implementation, and lead to the development of strategies to de-implement outdated healthcare practices.

### Argument for rethinking the role(s) of policy in D&I efforts

The fields of political science and public administration have repeatedly called on public health scientists to go beyond measuring the impact of a policy on health outcomes and intentionally examine how policy and politics influence the delivery of health services [18–22]. Doing so requires changes to how researchers conceptualize their work and the subsequent design and measurement choices that they make. Bernie and Clavier poignantly note that “health promotion research is inherently political” and caution researchers against conceptualizing a fictitious world where health interventions and scientific research are politically neutral topics [21]. For example, recent policy changes in the USA demonstrate strong political influence, some to the detriment of public health and individual rights through restrictions in access to care [23–25]. Public health is also criticized for naïvely perceiving policymaking as a linear process with research serving as the strongest influence over decision-makers [20, 21, 26, 27]. Policy D&I research has the potential to address these criticisms by examining how complex, nonlinear policymaking and implementation processes are shaped by a plurality of interests including evidence, politics, personal and societal values, finances, and other factors of variable transparency [14, 28–30]. Early health policy implementation research focused solely on the mid-implementation process [31]. Just as traditional D&I has increasingly focused on multiple phases of implementation [32], guidance is needed to support researchers in understanding health policy D&I decisions and activities across pre- and mid-implementation and sustainment phases. To address these critiques scientifically and practically, D&I research require theories, models, and frameworks (TMF) that are health policy conscious, that is, they meaningfully consider the dynamic nature of policy, polity structures, processes, political ideologies, and policymakers that shape implementation and sustainment.

D&I research emphasizes the importance of grounding studies in TMF to structure our understanding of how and why relationships between variables lead to certain outcomes [33]. Delineating a TMF to inform study approach is critical to developing a successful D&I research proposal [34–36]. In 2012, Tabak et al.’s review identified nine policy-level D&I TMF [37]. The online D&I models in health search now includes 26 TMF that address (albeit with varying degrees of depth) the socioecological level of policy in some way [38]. However, many of these TMF are political science models without clear D&I processes described, content area specific [39–41], center on a population or setting [42], merely describe implementation phase activities [31], and focus on systems-level policy only [5], which limits their generalizability and utility for conceptualizing the multifaceted roles that policy can play. D&I TMF that are topic, setting, and population agnostic, useful across pre- and mid-implementation and sustainment phases, capable of addressing the determinants of, and policy implementation processes that occur across systems or within organizations are necessary to advance knowledge about the role health policy can play as the evidence-based “thing” to be implemented or within a causal pathway leading to successful dissemination or implementation [43].

We have two options to advance policy D&I research: (1) create new policy D&I-specific TMF that meet the above criteria or (2) adapt existing TMF to better conceptualize policy. We argue that a dearth of generalizable, policy-conscious TMF is not sufficient cause for developing new, untested TMF. Instead, researchers should optimize existing D&I TMF to more accurately capture policymaking and implementation processes over time and consider how policy (and its inseparable politics, polity structures, and policymakers) impacts the delivery of evidence-based practices (EBPs). In this article, we propose and describe practical recommendations to adapt the Exploration, Preparation, Implementation, and Sustainment (EPIS) framework [32, 44] to meaningfully conceptualize policy, with the hope that researchers will apply these recommendations to enhance their empirical investigations with EPIS and other TMF.

## Systematic scoping review of policy D&I studies that employed the EPIS framework

We conducted a systematic scoping review, guided by the PRISMA checklist [45] (Additional file 1) to identify examples of policy D&I research that used the EPIS framework, with the goal of generating recommendations for improved TMF optimization.

EPIS was chosen as the focus of this review for four reasons. First, EPIS is an influential TMF in the D&I field, having been cited in more than 1600 publications. Second, EPIS is one of few D&I TMF that directly investigates the temporal nature of D&I activities and determinants over time, suggesting that D&I stakeholders begin the *exploration* phase of any initiative by assessing the need for change and ways to accomplish change. The decision to adopt a new practice or policy propels them into *preparation* phase activities until active *implementation* of new or modified services commence. Stakeholders graduate to the *sustainment* phase when they can shift resources from implementation to maintenance activities. EPIS acknowledges that temporal phases may be recursive as priorities, resources, and policies shift over time [46]. Third, EPIS is highly flexible and neutral in regard to topic, setting, population, and policy scope, making it a useful framework for investigating policy at different ecological levels and in diverse contexts [32, 44]. Fourth, EPIS includes domains (e.g., outer/inner context) and constructs found in other D&I TMF, which heightens the potential generalizability of recommendations presented below.

Most traditional political science conceptual models describe the temporal process of policymaking [47, 48], but do not provide insights on relationships between potential determinants and mechanisms across contexts. EPIS, like many D&I TMF (e.g., Consolidated Framework for Implementation Research; CFIR [49]), conceptualizes an *outer context* to describe a broad environment of influential factors, and an *inner context* comprised of local organizational factors that impact D&I efforts [32, 44]. These multi-level *outer* and *inner contexts* provide operational settings to investigate where a policy originates and exudes influence. In the 2011 EPIS introduction, Aarons et al. identified “sociopolitical/funding” as an *outer context* construct to acknowledge that legislative environments can influence stakeholders’ desire to explore potential changes and enhance or constrain available resources to adopt and sustain new practices [32]. A later iteration of EPIS reconceptualized policy’s influence as “service environment/policies” and “funding/contracting” [44]. The *innovation factors* domain is broadly defined, allowing for the study of one or more EBPs or policies [32, 44]. *Bridging factors* acknowledge the interrelated nature of *outer* and *inner contexts* and posit that specific structures, intermediaries, and activities (which can include policies and policy advocates) are needed to align contexts to support D&I success [50]. The flexibilities afforded across EPIS domains provide space to conceptualize the dynamic and multiple roles policy can play. These reasons led us to conclude that conducting a scoping review of policy research guided by the EPIS would yield useful lessons about optimizing TMF.

### Defining “policy” for the scoping review

Eligible studies employed EPIS to investigate the role of policy in D&I efforts. We defined “policy” and “policymaking” broadly using language from political science and public administration research. Policies are a series of interrelated decisions (e.g., legislation, rules) and purposive actions or inactions by decision-makers to execute agency goals [51]. Studies could acknowledge policies of any scope including “big P” policies like federal, state, county, and city laws, regulations and administrative rules designed by government agencies, or “little p” policies including organizational rules and professional guidelines. Articles were also included if they mentioned the role of policy or the influence of policymakers and politics (e.g., describing the “sociopolitical environment”) in the policy environment. We focused on health policies but adopted a Health in All Policies approach to consider how any policy, regardless of its intended focus on health or any other issue, can be strategic tools that influence social determinants of health and have a direct impact on population health outcomes [52]. Given the broad nature of scoping reviews, we did not exclude studies based on the topic (e.g., health, environment, taxes) of the EBPs/program/policy investigated, origin (outer or inner context), type (big P or little p), or influence of the policy addressed in a D&I effort.

### Search strategy and results

We first reviewed results from a recent systematic review of EPIS [44] that identified 67 articles published between 2011 (when the original framework was published) and May 2017 that used EPIS to guide dissemination or implementation efforts [44]. We obtained and reviewed the raw data from their systematic review to identify any articles that included policy considerations in their research. Simultaneously, we replicated Moullin et al.’s search criteria [44] (Additional file 2) in Web of Science, PsychINFO, and PubMed to identify more current relevant articles that were published between May 2017 and July 2022. Manual hand search methods were used to include relevant publications not yet indexed in databases.

Of the 1052 articles screened, 123 met criteria for full-text review. Most articles were excluded during the screening process because they cited the Aarons 2011 article but did not apply EPIS to their project (*n* = 719), or if they applied EPIS, they did not address policy in any way (*n* = 189). Articles were excluded after full-text review if they did not define any aspect of policy’s role in a D&I effort (*n* = 27). Ultimately, 96 articles were included in our qualitative synthesis (Additional file 3). Two researchers (ELC, RLH) performed screening and qualitative synthesis activities using a standardized scoping review template. We extracted data on policy characteristics (e.g., big P/little p), policy goals, the focal EBPs/policy, breadth and depth of EPIS use, and any policy-relevant adaptations to EPIS.

### Recommendation development process

Five D&I scientists reviewed the extracted data and engaged in a consensus decision-making process to develop recommendations for optimizing TMF for policy D&I research. The authors have expertise in developing (GAA is one of the EPIS developers), advancing, and adapting TMF [(e.g., EPIS, CFIR, Practical, Robust, Implementation and Sustainability Model (PRISM)] to study D&I efforts and political science topics in US and global public sector health and allied health systems, community pharmacy, and criminal justice settings. Grounded in qualitative thematic analysis methods [53], we initially organized data from the scoping review to compare how prior research conceptualized the role of policy, policymakers, or politics as an *outer* or *inner context* variable, an *innovation factor*, *bridging factor*, or other variable. ELC identified similarities and differences in these conceptualizations and noted when policy’s role was ambiguously defined. ELC presented the qualitative synthesis and preliminary TMF recommendations to the coauthors. Coauthors reviewed the data and recommendations and then expanded on the suggestions, added additional ideas, and asked questions about the extracted data and role of policy. Revisions to recommendations were made over a 1-year period in a sequential process while maintaining a log of edits to capture the consensus decision-making process. Discrepancies in recommendations were resolved through team discussions. This process resulted in development of six recommendations for optimizing TMF for policy D&I research.

## Recommendations for optimizing EPIS to investigate health policy D&I

We provide six recommendations to advance policy D&I research through EPIS optimization:

1. Specify dimensions of a policy’s function.
2. Specify dimensions of a policy’s form.
3. Identify and define the nonlinear phases of policy D&I.
4. Describe the temporal roles that stakeholders play in policy D&I over time.
5. Consider policy-relevant outer and inner context adaptations.
6. Identify and describe bridging factors necessary for policy D&I success.

**Fig. 1:**
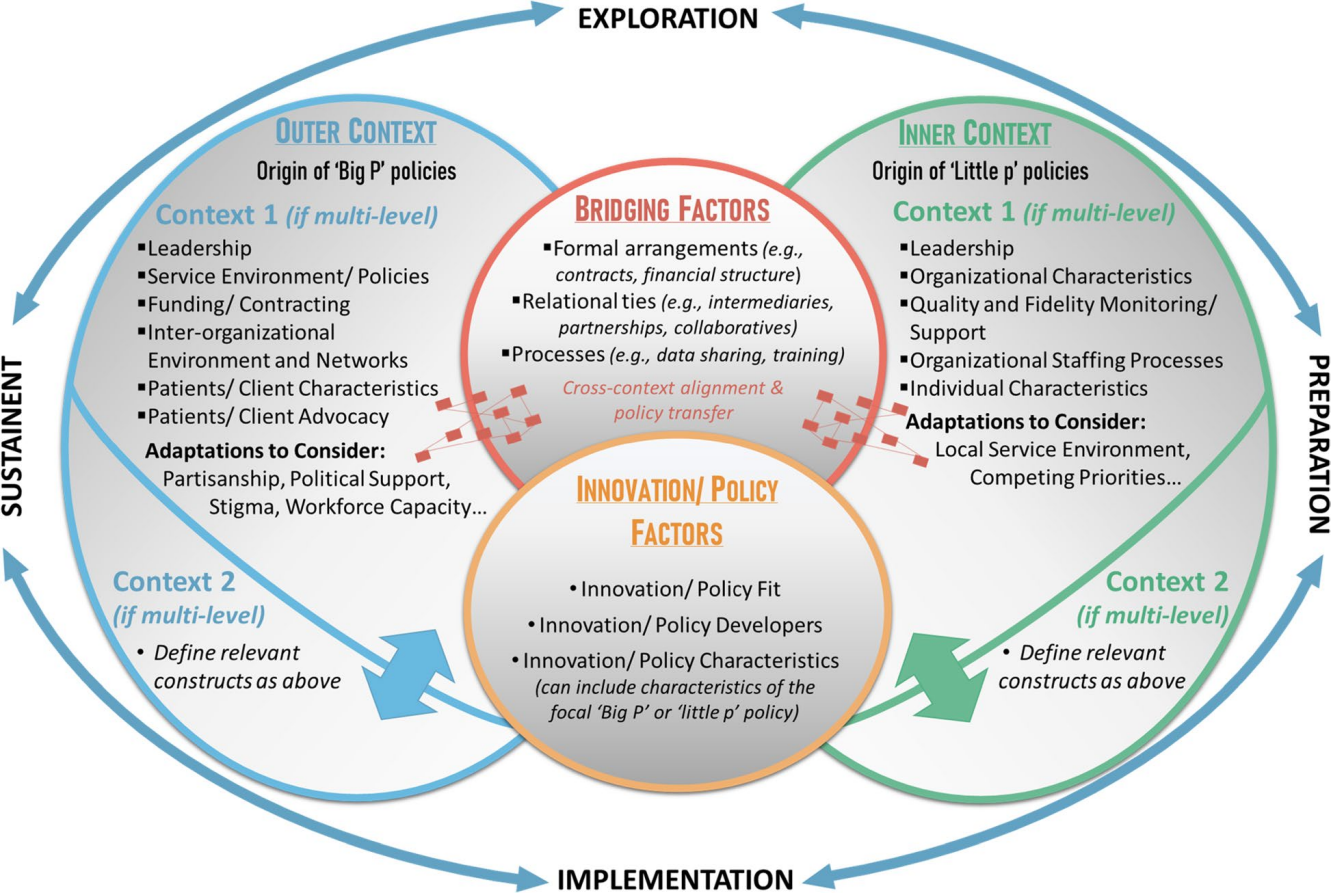
Policy optimized version of the Exploration, Preparation, Implementation, and Sustainment (EPIS) framework

Recommendations 1–2 optimize EPIS by defining key dimensions of a policy so that researchers can determine which domain/construct it should occupy and to understand where policy exists within a causal pathway. Recommendations 3–4 describe how researchers can use EPIS to conceptualize policy implementation activities over time and specify which policy-relevant stakeholders are represented in domains/constructs. Recommendations 5–6 acknowledge that existing domains/constructs may be underdeveloped for considering policy D&I factors and offer guidance for researchers to advance EPIS specification. Although recommendations are illustrated through EPIS application (Fig. 1) [32, 44], we provide examples of how they can be applied to other D&I TMF. We provide hypothetical research examples to illustrate the applicability of these recommendations to global settings, across different health topics, and roles of policy in D&I efforts.

### Recommendation 1: Specify dimensions of a policy’s function

Few D&I studies specifically investigated policy as the evidence-based thing or as a strategy to be tested. Most alluded to policy as a factor in a vaguely described *outer context* but did not report on its purpose. *Outer contexts* were described generally as the “public and broader policy context,” “community,” and “outer system level of a broader environment.” *Inner contexts* were more clearly defined as specific state agencies, school districts, or healthcare provider organizations. Few articles defined the domain constructs (e.g., leadership, service environment agencies, funders, advocacy groups) responsible for creating and implementing policy or who might benefit from its passage.

The first recommendation is to assess the policy’s function describing the fundamental purpose of a policy [50, 54]. Function dimensions include the following: (1) policy goal(s), (2) policy type, (3) context, and (4) capital exchanged. Specifying these attributes will help researchers determine what role(s) a policy plays in D&I success and which domain/construct it occupies. Researchers should first ask, “what is the goal or intent of this policy?” This recommendation echoes early policy implementation research which argued that correctly identifying policy goals is critical to determining whether implementation was successful [31]. Policies may aim to affect a broad or narrow scope of change or to formalize something that is already being done in practice. Policies with ambiguous goals may promote confusion around implementation activities and have little impact [31]. Researchers should review legislative documents, government and organizational strategies, press releases and news articles, conduct legal mapping studies [55], or key informant interviews to specify policy goals. A single policy may have one or multiple goals; researchers should determine which goal(s) are critical to their D&I effort. Specifying the policy goal will help clarify if the policy is the evidence-based intervention, an implementation strategy to promote adoption of an EBP/program, a mechanism (series of events that promote the success of another implementation strategy), a precondition (i.e., factor necessary to activate the mechanism), determinant (i.e., barrier, facilitator), mediator (i.e., a variable that intervenes on the relationship between the implementation strategy and outcome), or moderator (i.e., a variable that alters the influence of another implementation strategy) [43]. Lewis et al. (2018) provide a comprehensive description of these causal pathway terms, which can aid researchers in further identifying a policy’s goal [43]. Specifying the policy goal can also reveal outcomes of interest (see “Recommendation 2: Specify dimensions of a policy’s form”) from the policy D&I effort — thereby advancing new policy-relevant implementation effects beyond traditional D&I outcomes (i.e., acceptability, adoption, appropriateness, feasibility, fidelity, implementation cost, penetration, and sustainability) [56].

Researchers can then determine if the policy represents a “big P” or ‘little p’ policy type. Researchers can observe in-person or broadcasted hearings and/or document review of public policy records, policymaker meeting notes, white papers, and governmental strategies to help specify the functions of “big P” policies, although there may be many “behind the scenes” nuances to consider. Qualitative interviews with key informants may be needed to describe the function dimensions of “little p” policies if organizational documents (e.g., organizational strategy plans) are not publicly available. Researchers can investigate if/how “big P” policies turn into “little p” policies or vice versa over time.

Correctly identifying the policy goals and type will aid researchers in describing the *outer* and *inner contexts* where the policy originates and/or is implemented and potential implementation outcomes. The complexity of policymaking processes means that *outer* and *inner contexts* can be multi-level. The similarity in EPIS domain names makes this recommendation applicable to other TMFs including PRISM’s external/internal context [57] and CFIR’s outer/inner setting [49, 58]. Researchers should define all relevant policy contexts and levels to understand environmental factors that influence D&I processes. Finally, researchers need to identify the resources or capital exchanged (e.g., money, knowledge, data, training, political will) through the policy (then determine if those resources constitute a *bridging factor*, see “Recommendation 6: Identify and describe bridging factors necessary for policy D&I success”). Identifying the capital exchanged will help researchers understand why and when a policy is successfully implemented across multi-level contexts (i.e., “policy transfer”) [5].

Researchers need to specify a policy’s function to determine if their framework should include the policy of interest as an *outer/inner context* factor, *bridging factor*, implementation strategy, or as the *innovation factor*. This is critical because it will help guide researchers to hypothesize about potential contextual constructs and relationships that influence D&I processes and outcomes. An example scenario for applying Recommendation 1 is presented in Table 1.

**Table 1:** Example for specifying dimensions of a policy’s function (Recommendation 1)

**Example scenario**	A state health agency announces a policy that requires insurance companies and contracted providers to report on quality metrics related to the delivery of behavioral health services.
**EPIS framework application steps**	1. Review legal documents or conduct qualitative interviews with policymakers to specify the policy goal(s). • The policy goal is to increase the quality of behavioral health services delivered. 2. Review legal documents to specify the policy type as part of its *innovation characteristics* or to place it as a determinant or other variable within the EPIS framework. • The policy is a “big P” policy type because it arises from the state health agency and requires compliance from payors and providers. 3. Conduct a landscape analysis and/or speak with stakeholders about the policy to describe the multi-level outer and inner contexts affected by dissemination and implementation efforts and determine whether they are multi-level. • The state health agency is part of the *outer context.* • The multi-level *inner context* consists of insurance companies and provider organizations who must submit quality metric reports. • Further investigation is needed to better define the *outer* and *inner contexts* and their relevant stakeholders. EPIS constructs (e.g., leadership, organizational characteristics, service environment) should be investigated regarding the state health agency, insurance companies, and provider organizations. For example, is *outer context* leadership limited to the state health agency, or should it also include the governor or federal agency leaders? Is the service environment specific to publicly funded behavioral health services, or does this include privately funded care? 4. Review legal documents, policy budgets, and any investigative journalism published about the policy to identify any capital exchanged. • The capital exchanged via the policy is data on the quality of behavioral health services delivered. • Researchers should examine how frequently this capital is exchanged and which stakeholders stand to benefit from these data (e.g., patients deciding about care options, provider organizations seeking a competitive ranking, vendors paid to manage the reporting system).

### Recommendation 2: Specify dimensions of a policy’s form

Few D&I studies have investigated policy as the evidence-based intervention to be implemented or as the implementation strategy. As a result, policy developers, their decision-making processes, and policy components are infrequently defined in D&I articles. To better conceptualize policy, researchers should clearly define the policy’s form: (1) its origin and creators, (2) structural components, (3) dynamism, and (4) (un)intended outcomes. Specifying a policy’s form will reveal the structures and processes that influenced how the policy was developed and can guide empirical research measuring how specific policy characteristics influence D&I outcomes [50]. Knowledge about policy structure (i.e., what it specifically enforces) can help researchers investigate which role policy plays in a causal pathway for D&I efforts and where it should be placed in the TMF (e.g., *outer/inner context*, *innovation factor*).

Policy origin refers to how the policy was developed and the stakeholders involved in its creation. For example, was the policy developed by agency staff, an expert workgroup, via a collaborative process with the public or advocacy groups? Understanding the origin story creates transparency in the policymaking process [11] to reveal the nature of “evidence” (e.g., research vs. personal beliefs) used to inform decisions and the types of interests represented during policy development. Social network analysis can aid in identifying actors involved in the policy’s creation.

If policy is the evidence-based “thing” to be implemented, the EPIS *innovation factors* domain can be specified. In other TMFs, researchers can specify policy within the innovation [49, 58], evidence [59], or intervention domain [57]. EPIS’ “innovation developers” construct can help define the policy’s origin. But policies might serve another role (e.g., as a determinant), and specifying where the policy developers reside (i.e., in *outer* or *inner contexts* and whether partisanship is part of the policies’ impetus) and their networks of influence can be useful to understanding which stakeholders need to be strategically engaged in the D&I effort or be the target of D&I strategies. For example, Purtle et al. identified US state legislators as a target group involved in policy decisions that impact children’s exposure to adverse childhood events (ACEs) [60]. They found that democratic policymakers were more likely to engage with dissemination strategies that included projected lifetime costs to the public system associated with every nonfatal ACE case, while economic data did not alter republican’s engagement on this policy issue [60].

Specifying the policy structure requires asking whether the policy is enforceable or effective enough to impact implementation. Researchers should determine if the policy represents a funded or unfunded mandate, suggested guidelines, or some other structure that will impact the urgency and compliance of stakeholders. Document review of the policy itself should clarify structural components. Informational interviews with policy developers can also yield insights on policy structures.

Dynamism describes the policy’s intent and potential for permanence. Researchers should investigate if the policy has an expected lifetime (e.g., 5-year demonstration project). Time-limited policies may have temporary political/public support that diminishes over time, ultimately leading to the policy’s dissolution. For example, COVID-19 mask mandates were commonly implemented as time-limited policies that increasingly generated public backlash mounting political pressure on politicians and public health agencies to prevent mandate renewal [61]. Policies without time limitations can face competing or supporting policies over time, political pressure, or advocacy from the *outer* and *inner contexts* that influence policy longevity. Researchers can investigate a policy’s dynamism by using legal mapping methods [55] or document review including white papers, government or organizational reports, legal, news, and social media sources. The prevalence of siloed health agencies [62, 63] suggests that competing or complementary health policy implementation efforts and political support exist, and qualitative interviews can help explain how these factors impact dynamism of the focal policy. Longitudinal media analyses and public opinion survey data can reveal how support for a policy changes over time and influences its permanence.

Identifying or measuring the intended and unintended outcomes of policy implementation represents the final form dimension. Policy outcome measurement can be the primary research aim or contribute to understanding the policy D&I process. For example, Crable et al. investigated implementation strategies used by Medicaid policymakers’ to encourage substance use treatment providers to adopt EBPs during each EPIS phase [7]. Citing policy reach and fidelity outcomes from state evaluation projects helped contextualize the impact of implementation strategies used in Preparation and Implementation phases [7]. Public testimony from constituents, advocacy groups, and lobbying firms can reveal potential unintended outcomes of policy implementation for researchers to investigate. Researchers should consider whether a policy is generating upstream and downstream outcomes and across which contexts. Upstream outcomes include the use of research evidence in policymaking and the overall fit of a policy with contextual factors. Downstream outcomes include how the evidence-based policy impacts quality, access, equity, and costs — which can be measured using large population health surveys or claims data. Qualitative descriptions and quantitative measures can be used to examine policy outcomes, and this methodological area is ripe for advancement [64, 65].

In EPIS, the *innovation factors* domain is commonly used to examine the developers, characteristics, and fit of an EBPs but can easily be adapted to investigate policy forms. Researchers should use “innovation developers” to describe the policy’s origin story, “innovation characteristics” to reveal its structure and its dynamism, while “innovation fit” describes the (un)intended consequences of a policy and its overall fit with contextual factors (Table 2). Policy forms can similarly be specified in RE-AIM/PRISM fit considerations regarding intervention/policy components or the overarching issues domain where policy representativeness, reasons, costs, benefits, and value can be defined [57]. In CFIR, researchers can adapt the innovation domain to specify policy forms including its source (i.e., origin). Trialability, adaptability, and complexity can reveal the potential dynamism, and cost informs one outcome [58]. Regardless of TMF used, researchers should specify if policy outcomes occur in *outer* and/or *inner contexts*.

**Table 2:** Example for specifying dimensions of a policy’s form (Recommendation 2)

**Example scenario**	The World Health Organization (WHO) publishes a Model List of Essential Medicines every 2 years as a guide for countries to use when defining their own essential medicines list. A National Health Insurance Program adopts the WHO Model List of Essential Medicines to define which medicines are available to citizens under the national health plan.
**EPIS framework application steps**	1. Specify the policy’s origin by describing the “innovation developers” construct. Note whether innovation developers arise from the *outer* or *inner context* (see Recommendation 1). • The innovation developers are the WHO Expert Committee on Selection and Use of Essential Medicines, who originate in the *outer context.* • Further investigation is needed to reveal which in-country Ministry of Health stakeholders were involved in the decision to adopt the WHO policy. 2. Review legal documents to specify the policy’s structure as part of its *innovation characteristics.* • While the Model List of Essential Medicines is originally structured as a recommended guideline, a country’s adoption of this list for their national health insurance program transforms it into a law that must be adhered to until it is changed. 3. Review legal documents or conduct interviews with Ministry of Health decision-makers to assess the policy’s dynamism as part of its *innovation characteristics.* • WHO’s biennial updating of the model list suggests that the in-country law may be semi-permanent. 4. Use mixed methods to identify outcomes of policy implementation including the policy’s overall *innovation fit.* • Researchers can investigate the cost of adopting the model list on their health system (e.g., are the costs of drugs on this list aligned with the country’s existing budget for these medicines?). • Researchers can investigate the alignment between drugs included on the model list and the country’s disease burden.

### Recommendation 3: Identify and define the nonlinear phases of policy D&I across contexts

Like policymaking, D&I processes are not linear [32, 59, 66, 67]. Our scoping review revealed few studies that examined D&I efforts across multiple EPIS phases. Most research focused on *Implementation* phase activities with little to no attention to how policy initially influenced or later modified implementation activities. Studying the nonlinear nature of policymaking and implementation processes is critical to understanding how and why evidence-based policies are adopted [21, 26].

Researchers should identify and define the nonlinear phases of policy D&I (Table 3). This process may require drawing different construct operationalizations within EPIS phases since contextual factors can yield different levels of influence and interaction over time. Researchers should identify the activities and stakeholders that characterize each D&I phase. Researchers can use EPIS phases or generic pre-, mid-, and post-implementation language to benchmark policy D&I activities. EPIS is particularly well-suited for achieving this recommendation given its temporal *exploration*, *preparation*, *implementation*, and *sustainment* phases and their dynamic relationship with other framework constructs. Researchers could integrate the use of group model building methods like causal loop diagrams to describe the role of policy over time, where reinforcing loops to indicate D&I momentum and balancing loops indicate stagnation [68]. Causal loops might vary depending on the EPIS phase in which they are proposed to occur. Mixed methods can further illuminate the stories behind causal loop diagrams to reveal contextual factors that motivated each phase.

**Table 3:** Example for identifying and defining the nonlinear phases of policy D&I across contexts (Recommendation 3)

**Example scenario**	Hospital leaders identify a high number of hospital-acquired infections in their patient population. Hospital leaders identify poor hand hygiene as a root cause of elevated infection rates and decide to adopt the Center for Disease Control’s Clean Hands Count campaign as a hospital policy. The hospital emails staff about the new policy, places soap and hand sanitizer in every patient room and hallway, displays posters reminding staff to clean their hands before and after entering each patient’s room, and incorporates hand hygiene education into staff meetings and provider rounds. Observers record hospital staff’s handwashing behavior and monitor the rate of hospital-acquired infections over time.
**EPIS framework application steps**	1. Organize documented policy dissemination and implementation activities by time-bound EPIS phases. • *Exploration*: Hospital leaders examined the need for change by reviewing data on hospital-acquired infection rates, identify hand hygiene as a problem, and hospital leaders decided to adopt the Clean Hands Count campaign as hospital policy. • *Preparation*: Hospital leaders designed implementation strategies to facilitate adoption of the Clean Hands Count initiative. • *Implementation*: Policy notices, hand hygiene supplies placement, and educational and reminder strategies were used. Observers monitored fidelity to the hospital policy and corresponding implementation strategies. • *Sustainment*: Over time, if infection rates decrease and employees continue hand hygiene protocols, the hospital may enter *sustainment*, whereby hand hygiene becomes part of the hospital’s culture and workflows. 2. Use mixed-methods approaches to investigate the contextual factors that propel the hospital to the next phase or influence them to revisit activities from past phases. • Examine how the hospital leaders maintain focus on hospital acquired infection rates or if leadership changes impacted this policy focus over time. • Examine any resource changes or competing demands that amplified or diminished focus on this policy over time.

### Recommendation 4: Describe the temporal roles that stakeholders play in policy D&I over time

Recommendation 3 highlights the need to understand how *outer* and *inner contexts* change over time, while Recommendation 4 advises researchers to specifically investigate how stakeholder roles and responsibilities in these contexts change over time. While some articles included in this review mentioned policy as a determinant of D&I efforts, they seldom described specific *outer context* “leadership” such as government officials charged with shaping or enforcing policy. “Interorganizational networks” of stakeholders were more frequently identified as having some distal influence over D&I processes, but their roles as implementation partners or intermediaries facilitating implementation efforts were not discussed. Several articles focused on the role that *inner context* “leadership” played in prioritizing and directing implementation efforts. Fewer articles addressed the role of stakeholders’ “individual characteristics” influencing implementation efforts.

Stakeholders involved in policy D&I efforts can enter, exit, and change positions over time. Researchers should document these positions, responsibilities, and movements in their framework to understand who is making decisions about policy development, dissemination, and implementation. Researchers can start by identifying the *outer* or *inner context* “leadership.” In addition, the role of *outer context* “interorganizational networks,” “advocacy groups,” “clients/patients,” and *inner context* frontline implementers as well as “intermediaries” who support the implementation of policy across contexts should also be considered. Some stakeholders will be involved throughout the entire policy lifetime (e.g., *exploration*, *preparation*, *implementation*, *sustainment*) or during time-limited phases where they make strategic contributions. Researchers should optimize their TMF to conceptualize the influence of all relevant stakeholders across *outer* and *inner contexts* and to determine if they serve in a *bridging factor* role, such as an “intermediary” aiming to align *outer* and *inner contexts* to promote policy implementation. EPIS includes multiple constructs describing stakeholders across domains, enabling researchers to capture how these roles change over time. If using other TMF, we recommend detailing individuals involved [58], specifically who is facilitating [59] policy D&I processes and the representativeness of stakeholders [57].

To conceptualize stakeholders’ roles over time, researchers can draw multiple time-bound versions of their EPIS framework. For example, researchers can specify stakeholder roles and responsibilities in *outer* and *inner contexts*, or as *bridging factors* during the *exploration phase*, and then re-specify those roles for the *preparation phase* to see which elements changed over time. Researchers can use multiple data collection methods to identify stakeholders including policy and meeting document review, social network analysis, ethnographic observation, stakeholder surveys, and qualitative interviews. Snowball sampling techniques [53] can reveal unexpected stakeholders across phases. An example scenario for applying Recommendation 4 is provided in Table 4.

**Table 4:** Example for describing the temporal roles that stakeholders play in policy D&I over time (Recommendation 4)

**Example scenario**	The Ministry of Health is proposing policy that would legalize overdose prevention centers to provide a sanctioned, safe space for individuals to consume personal drugs in a medically supervised setting. A coalition of harm reduction organizations lobby for the adoption of this policy by providing research that demonstrates that overdose prevention centers are associated with increased rates of initiation and engagement in substance use treatment, reduced rates of fatal and nonfatal overdose, and reduced rates of HIV and hepatitis C transmission. Once the law is passed, the coalition of harm reduction organizations and a local hospital help to implement by allocating trained staff for overdose prevention centers using their existing resources.
**EPIS framework application steps**	Determine which EPIS phases are relevant to this dissemination and implementation effort (see “Recommendation 3: Identify and define the nonlinear phases of policy D&I across contexts”). • This scenario focuses on the *exploration*,* preparation*, and *implementation* phases. 1. Conduct document review, ethnographic observation, network analysis, surveys, or interviews to identify which Ministry of Health stakeholders were acutely involved in each phase. • Identify Ministry of Health stakeholders who were part of the adoption decision during the *exploration* phase. • Identify stakeholders tasked with assessing the potential barriers and facilitators of an overdose prevention center during the *preparation* phase. • Identify which stakeholders were involved in *implementation* of the policy and creation of the overdose prevention center. 2. Use qualitative methods to describe factors that influence specific stakeholder behavior in each phase and whether those roles change over time. • Describe which factors led policymakers to *explore*, *prepare*, and *implement* policy legalizing overdose prevention centers. • Investigate how the coalition of harm reduction organizations originally served as an intermediary *bridging factor*, providing evidence to policymakers during the *preparation* phase. • The harm reduction organizations and the hospital became *inner context* frontline implementers during the *implementation* phase after the law was passed.

### Recommendation 5: Consider policy-relevant outer and inner context adaptions

TMF should guide the translation of research into policy and practice and elucidate and explain the relationship between contextual determinants, D&I strategies, and outcomes [33, 36]. Existing TMF present an incomplete organization of factors that impact policy D&I. Very few studies in the EPIS scoping review examined how specific policymakers (i.e., not just “leadership”), political institutions (i.e., polity structures), and politics played a role in D&I efforts. Most articles in the scoping review used a fraction of the EPIS constructs within *outer* and *inner contexts*, *bridging factors*, and *innovation factors* domains. Some articles did make adaptations to *outer* and *inner contexts* (Additional File 4). We argue that researchers should incorporate and define new policy-conscious constructs, as needed, to better understand the studied context or test new hypotheses about policy D&I processes, relationships, and causal pathways (Table 5). However, researchers should be careful not to include an unwieldly number of constructs that hinders meaningful investigation of the relationships between each.

**Table 5:** Example for considering policy-relevant outer and inner context adaptions (Recommendation 5)

**Example scenario**	A community health center implements an organizational policy stating that all clients will be offered free HIV testing and counseling services during every clinic visit.
**EPIS framework application steps**	1. Specify the type of policy and context where it will be implemented (see recommendations 1 and 2) to determine which existing constructs are most relevant. • The community health center represents the *inner context* where implementation of this “little p” policy will occur. • The *inner context* “individual characteristics” construct describes the influence of attitudes toward HIV testing and counseling services that impact implementation success. 2. Ask stakeholders or conduct literature and document review to reveal additional contextual factors might influence policy implementation. • Given the historical stigma surrounding HIV, researchers may want to include another construct within the EPIS *inner context* to measure “provider stigma” toward persons living with HIV. • The *outer context* might be adapted to include “societal stigma” about HIV, hesitation to participate in HIV testing and counseling services.

Researchers should conduct literature reviews and speak with stakeholders in the study setting to identify relevant TMF adaptations that are necessary to conceptualize policy and guide empirical research. Potential adaptations to EPIS’ *outer context* include adding constructs like “political support” (to address partisanship), “societal stigma” (toward an issue or population targeted by the policy), “workforce capacity” (if implementing a policy that impacts provider responsibilities), and “news and social media attention” (which can sway societal and political support for a policy). Researchers should consider *inner context* adaptations which can include defining an organization’s “local service environment” (to describe how the existing service array might change due to policy D&I efforts). Adapting EPIS and other TMF to include relevant contextual influences helps to reveal new relationships between D&I strategies, mediating and moderating factors, and mechanisms that produce both desired and unintended outcomes [43].

### Recommendation 6: Identify and describe bridging factors necessary for policy D&I success

*Bridging factors* represent structures, relationships, intermediaries, and processes that support *outer-inner context* alignment, policy transfer, and D&I success [44, 50, 69]. Like stakeholders, *bridging factors* may be omnipresent throughout all phases of dissemination or implementation or have a time-limited role [7, 50]. Although “bridging factors” language is specific to EPIS, these alignment enhancing factors can be conceptualized as domain-spanning linkages in other D&I TMF (e.g., boundary spanners that work across contexts to promote implementation outcomes). Recent research describes how contracts [69] and renegotiated reimbursement rates [7] between government agencies and clinical service providers are formal structures that function as *bridging factors*. Relational ties, like partnerships between government agencies and provider organizations, can also represent *bridging factors*. Stakeholders (e.g., lobbyists, consultants, advocates) who support the passage of a policy in the *outer context* and its implementation in the *inner context* serve in *bridging factor* roles [11, 50]. Researchers should investigate personal (e.g., financial) and professional (e.g., influence) gains individuals receive from serving as a *bridging factor*. Data and information sharing processes between *outer* and *inner context* entities (e.g., measurement-based care reporting) can also serve as *bridging factors* to promote cross-context alignment [69, 70] or policy transfer. Despite the important role *bridging factors* serve in achieving D&I success, their functions and forms are significantly understudied, and few studies in the scoping review enhanced our knowledge of their capacity to activate change.

Researchers should investigate and describe the presence or absence of necessary *bridging factors* for policy D&I success (Table 6). Such research would augment knowledge about how “big P” policies transfer from the outer to the inner context, how inner context “little p” policies are spread to the outer context, and how policies can diffuse across contextual levels [71–73]. Researchers can use qualitative methods to ask key informants about the nature and utility of structures, relationships, intermediaries, and processes supporting *outer-inner context* alignment and policy transfer processes. Snowball sampling techniques and social network analyses can help identify intermediaries and relational ties critical to policy implementation. Asking questions about how evidence is used to inform policymaking or how a policy is implemented can reveal when formal structures or processes serve as *bridging factors*.

**Table 6:** Example for identifying and describing bridging factors necessary for policy D&I success (Recommendation 6)

**Example scenario**	Researchers are studying local Board of Education policymakers’ use of evidence when designing school district immunization requirements for meningococcal disease. The national disease control agency and several professional medical societies recommend meningococcal vaccination for all school-age children. A parental advocacy group stages a local protest against vaccine requirements.
**EPIS framework application steps**	1. Specify the contexts being investigated to understand the stakeholders involved (see recommendations 1 and 4). • The government agency, professional medical societies, and advocacy group all operate in the *outer context.* • The Board of Education represents the highest level of the *inner context* with individual schools in the board’s district operating at lower levels of the *inner context.* 2. Conduct interviews, attend public meetings to determine whether either of the *outer context* entities (i.e., professional medical societies, parental advocacy group) evolve as “intermediary” *bridging factors* by directly providing Board of Education policymakers with information about the pros or cons of the proposed immunization policy. • Examine the type of information or “capital exchanged” (e.g., scientific evidence about vaccine safety, misinformation, personal beliefs) being shared by “intermediaries”. • Determine which phase(s) (i.e., *exploration*, *preparation implementation*, or *sustainment*) intermediaries play an active role delivering information/capital. • Investigate how the presence of a *bridging factor*, like a professional medical society, influences how immunization requirements are defined in policy, disseminated to parents, and implemented in practice.

## Conclusion

TMF shape how researchers conceptualize studies, determine which variables will be measured, and where, when, and how EBPs and strategies are employed. Existing TMF do not sufficiently address health policy’s role in D&I, which limits advancement of this important field of research. Instead, policy-relevant constructs are frequently absent or treated as nuisance variables [5, 18, 19]. Table 7 summarizes six recommendations to help researchers improve how policy-relevant factors are conceptualized in EPIS so that empirical studies are better positioned to test and explain causal pathways that support the use of evidence in policymaking and the implementation of evidence-based policies. Advancing policy D&I research does not require “reinventing the wheel” with new TMF. Instead, researchers should apply these recommendations to EPIS and other TMF to define health policy’s role in D&I efforts and advance empirical policy D&I research. Enhanced specification of health policy’s role may support future work defining health policy D&I outcomes beyond those measured in traditional D&I efforts (i.e., acceptability, adoption, appropriateness, feasibility, fidelity, implementation cost, penetration, and sustainability) [56]. These TMF recommendations are not static but can serve as guidance for the growing body of policy-focused D&I. Future work should also investigate how these recommendations support calls for integrating D&I, public policy, and knowledge translation [5] to understand policy implementation processes outside of health and healthcare settings (e.g., criminal justice reform, housing and community development policies).

**Table 7:** Six recommendations for optimizing TMF for D&I policy use

Recommendation	Considerations for each recommendation
1. Specify dimensions of a policy’s function.	• Identify the policy goal(s). • Identify the policy type (i.e., big p or little p). • Describe the affected multi-level outer and inner contexts. • Identify capital exchanged.
2. Specify dimensions of a policy’s form.	• Identify the policy’s origin. • Describe the policy’s structure. • Assess the policy’s dynamism or permanence. • Identify the (un)intended outcomes of policy implementation.
3. Identify and define the nonlinear phases of policy D&I across outer and inner contexts.	• Describe temporal benchmarks for policy dissemination and implementation processes.
4. Describe the temporal roles that stakeholders play in policy dissemination and implementation over time.	• Identify key stakeholders (e.g., politicians and/or agency staff who create or influence policy, lobbyists, advocates) and their responsibilities at each defined temporal benchmark.
5. Consider policy-relevant outer and inner context adaptations.	• Incorporate relevant outer/inner context constructs that are not included in existing frameworks to better explain potential determinants and mechanisms of dissemination and implementation efforts.
6. Identify and describe bridging factors necessary for policy dissemination and implementation success.	• Identify and describe the structures, relationships, and processes (i.e., bridging factors) that span outer and inner contexts to enhance policy dissemination or implementation.

These recommendations are designed to build on each other, resulting in optimal specification of EPIS for policy D&I research. Researchers may be hesitant to consider every activity described within each recommendation or to apply all six recommendations in one study. Researchers should consider how each recommendation will impact the quality and scope of their study. Additionally, these recommendations advance a growing set of tools [36, 44, 50, 69] for researchers to test and advance EPIS in D&I efforts.

## Supplementary Information

**Additional file 1. **PRISMA Checklist Applied to Scoping Review. Word document displaying how the PRISM checklist was applied to this narrative review.

**Additional file 2. **Search String for the Systematic Scoping Review. Word document listing the search terms used for the systematic scoping review.

**Additional file 3. **Flow Diagram of Search Strategy and Article Selection for the Scoping Review. Word document displaying a flow diagram summarizing the systematic scoping review search results.

**Additional file 4. **Identified Additions to Outer and Inner Context Domains in the Exploration, Preparation, Implementation, and Sustainment Framework from the Scoping Review. Word document displaying a table of adaptations applied the outer and inner contexts of the EPIS framework.

## Data Availability

Data sharing is not applicable to this article as no datasets were generated or analyzed during the current study.

## Abbreviations

D&I: Dissemination and implementation
EPIS: Exploration, Preparation, Implementation, and Sustainment framework
TMF: Theories, models, or frameworks
WHO: World Health Organization
