# Supplementary material for: Where is “policy” in dissemination and implementation science? Recommendations to advance theories, models, and frameworks: EPIS as a case example
Source: Implement Sci. 2022 Dec 12;17:80. doi: 10.1186/s13012-022-01256-x (PMC9742035; doi:10.1186/s13012-022-01256-x)
Supplement: Supplementary file 2 — Additional file 2. Search String for the Systematic Scoping Review. Word document listing the search terms used for the systematic scoping review. [file 13012_2022_1256_MOESM2_ESM.docx]

**Additional File 2. Search String for the Systematic Scoping Review**

*Article title used in searches:* Advancing a Conceptual Model of Evidence-Based Practice Implementation in Public Service Sectors

Web of Science: (TITLE AND THE “TIMES CITED” LINK FOR FULL LIST OF CITATIONS OF THE ORIGINAL EPIS PAPER)

PsychInfo: TITLE in REFERENCE and PEER REVIEWED

PubMed: single citation matcher (TITLE in ALL FIELDS CITED by), Cited In for PMID: 21197565
