# Supplementary figures and images for: Where is “policy” in dissemination and implementation science? Recommendations to advance theories, models, and frameworks: EPIS as a case example
Source: Implement Sci. 2022 Dec 12;17:80. doi: 10.1186/s13012-022-01256-x (PMC9742035; doi:10.1186/s13012-022-01256-x)

**Additional File 3. Flow Diagram of Search Strategy and Article Selection for the Scoping Review**


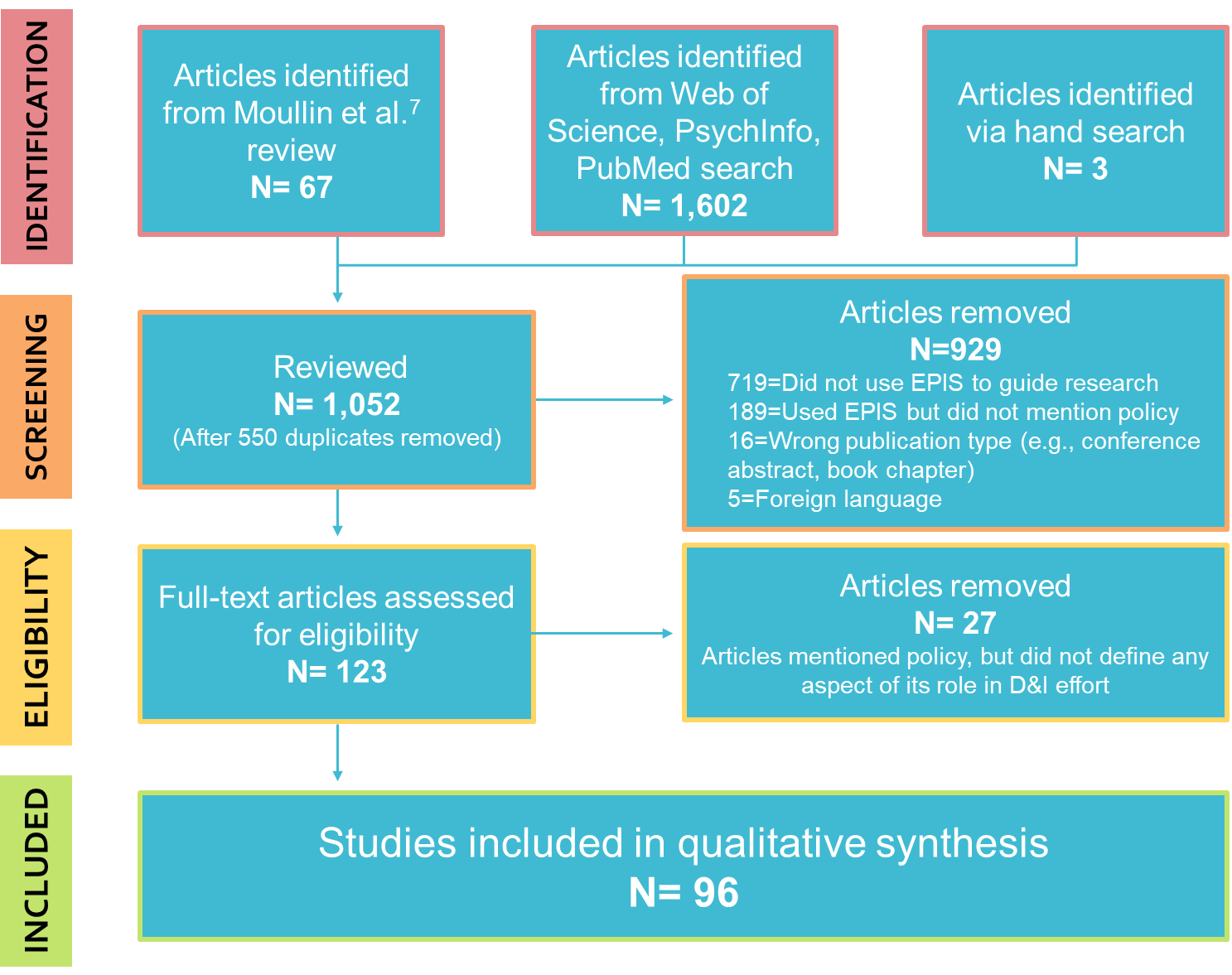

Supplement: Supplementary file 3 — Additional file 3. Flow Diagram of Search Strategy and Article Selection for the Scoping Review. Word document displaying a flow diagram summarizing the systematic scoping review search results. [file 13012_2022_1256_MOESM3_ESM.docx]
