# Supplementary material for: Where is “policy” in dissemination and implementation science? Recommendations to advance theories, models, and frameworks: EPIS as a case example
Source: Implement Sci. 2022 Dec 12;17:80. doi: 10.1186/s13012-022-01256-x (PMC9742035; doi:10.1186/s13012-022-01256-x)
Supplement: Supplementary file 4 — Additional file 4. Identified Additions to Outer and Inner Context Domains in the Exploration, Preparation, Implementation, and Sustainment Framework from the Scoping Review. Word document displaying a table of adaptations applied the outer and inner contexts of the EPIS framework. [file 13012_2022_1256_MOESM4_ESM.docx]

**Additional File 4. Identified Additions to Outer and Inner Context Domains in the Exploration, Preparation, Implementation, and Sustainment Framework from the Scoping Review**

| **EPIS Domain and Constructs** | **Construct additions and Adaptations Identified in Prior Research** |
| --- | --- |
| Outer Context | Absorptive capacity (an organization’s ability to recognize the value of, assimilate and apply new information) |
|  | Climate and culture of outer context organizations or agencies  *These constructs are typically assessed within the ‘organizational characteristics’ of the EPIS Inner Context* |
|  | Geographic issues (e.g., geographic variation in policy/services, distance between entities, need for transportation) |
|  | Fidelity monitoring  *‘Quality and fidelity monitoring/support’ is typically assessed within the EPIS Inner Context* |
|  | Legislative or administrative costs |
|  | News or press |
|  | Social hierarchy of professional disciplines interacting in the outer context |
|  | Social media |
|  | Political support or political will |
|  | Politics or partisanship |
|  | Prior implementation experience  *This construct is typically assessed as part of the ‘organizational characteristics’ or ‘individual characteristics’ of the EPIS Inner Context* |
|  | Public relations |
|  | Stigma |
|  | Workforce capacity |
| Inner Context | Client factors  ‘*Patient and client characteristics’ are typically assessed in the EPIS Outer Context* |
|  | Competing priorities within the organization |
|  | Cost of the evidence-based practice, program or policy to an organization |
|  | Geographic features or catchment area of an organization |
|  | Healthcare delivery model and available services |
|  | Prior implementation experiences  *This construct is typically assessed as part of the ‘organizational characteristics’ or ‘individual characteristics’ of the EPIS Inner Context, but may be called out as a separate construct within a multi-level contextual environment* |
